# Fluorescence‐based analysis of the intracytoplasmic membranes of type I methanotrophs

**DOI:** 10.1111/1751-7915.13458

**Published:** 2019-07-01

**Authors:** Kyle T. Whiddon, Ravindra Gudneppanavar, Theodore J. Hammer, Destiny A. West, Michael C. Konopka

**Affiliations:** ^1^ Department of Chemistry The University of Akron Akron OH USA; ^2^ Department of Polymer Science The University of Akron Akron OH USA

## Abstract

Most methanotrophic bacteria maintain intracytoplasmic membranes which house the methane‐oxidizing enzyme, particulate methane monooxygenase. Previous studies have primarily used transmission electron microscopy or cryo‐electron microscopy to look at the structure of these membranes or lipid extraction methods to determine the per cent of cell dry weight composed of lipids. We show an alternative approach using lipophilic membrane probes and other fluorescent dyes to assess the extent of intracytoplasmic membrane formation in living cells. This fluorescence method is sensitive enough to show not only the characteristic shift in intracytoplasmic membrane formation that is present when methanotrophs are grown with or without copper, but also differences in intracytoplasmic membrane levels at intermediate copper concentrations. This technique can also be employed to monitor dynamic intracytoplasmic membrane changes in the same cell in real time under changing growth conditions. We anticipate that this approach will be of use to researchers wishing to visualize intracytoplasmic membranes who may not have access to electron microscopes. It will also have the capability to relate membrane changes in individual living cells to other measurements by fluorescence labelling or other single‐cell analysis methods.

## Introduction

Methanotrophic bacteria are attractive candidates for bioremediation as well as industrial applications such as the production of bioplastics or other value‐added compounds. It is their natural ability to convert single‐carbon molecules into more complex, multi‐carbon compounds that have brought interest in using them as a powerful biological tool for converting methane into valuable compounds. Many methanotrophs also contain high lipid concentrations (Murrell, 2010) in the form of intracytoplasmic membranes (ICMs) which could be converted into biofuels. The formation of ICMs is tied to methane oxidation in methanotrophs (Trotsenko and Murrell, 2008). ICMs house one of the primary methane‐oxidizing enzymes, particulate methane monooxygenase (pMMO). To date, only one methanotrophic strain has been discovered to be lacking in pMMO and therefore ICMs (Vorobev *et al*., 2011), showing the importance of these structures in the function of these bacteria. Expression of the copper‐containing metalloenzyme pMMO is increased when more copper is available, and copper levels have also been found to modulate the extent of ICM formation (Hyder *et al*., 1979; Brantner *et al*., 1997; Dunfield *et al*., 2007). The ICMs of type I methanotrophs have been characterized as stacks of vesicular discs, as compared to those of type II methanotrophs where the ICMs are aligned along the periphery of the cell (Hanson and Hanson, 1996). Early observations by both electron microscopy (Whittenbury *et al*., 1970; De Boer and Hazeu, 1972; Brantner *et al*., 2002) and cell fractionation (Brantner *et al*., 2000) suggested that the ICMs of type I methanotrophs exist as invaginations of the cytoplasmic membranes.

A better understanding of methanotrophs and ICMs is required before many of the potential industrial applications for these bacteria would become practical (Jiang *et al*., 2010; Wendlandt *et al*., 2010). Knowledge of these structures has largely been limited to biochemical information learned from isolations through cell lysis techniques or structural information from transmission electron microscopy (TEM) or cryo‐electron microscopy (cryo‐EM) (Brantner *et al*., 1997, 2002; Kip *et al*., 2011; Wu *et al*., 2012; Tavormina *et al*., 2017). The development of rapid preparation methods, such as cryofixation and freeze substitution, helped to speed experiments so they could be completed in a day as opposed to longer times with more traditional methods (McDonald and Webb, 2011; McDonald, 2014). Such approaches also led to the development of methods where rapid fixation of samples would enable comparisons of cells from different times to look for dynamic changes. While TEM and cryo‐EM resolution is unparalleled, the current costs of purchasing and maintaining the required equipment may limit the availability to some researchers (Nogales and Scheres, 2015). In particular for cryo‐EM, the resources required for processing the images can be quite large or inaccessible to some potential users (Thompson *et al*., 2016). Electron microscopy by nature of fixing cells also prevents researchers from gaining information about the *in vivo* dynamics of ICMs in the same cell over time. Such data could conceivably be applied with other live‐cell labels to track dynamics during transitions in conditions.

To help remedy some of these issues, we have developed an approach using fluorescence microscopy to visualize ICMs in type I methanotrophs. It allows for the rapid acquisition of dynamic, whole cell information with minimal preparation time and sample perturbation. Fluorescent lipophilic dyes can rapidly label ICMs in type I methanotrophic bacteria so that it is possible to image and characterize hundreds of cells at one time. The results are consistent with the copper dependence of ICM formation and closely match results from TEM images. For this study, the type I methanotroph *Methylotuvimicrobium alcaliphilum* comb. nov. 20Z, previously *Methylomicrobium alcaliphilum* 20Z (Kalyuzhnaya *et al*., 2008; Orata *et al*., 2018), was the primary organism tested, although the methodology is broadly applicable to other type I methanotrophs.

## Results

### Staining of ICMs in type I methanotroph with FM dyes

**Figure 1:**
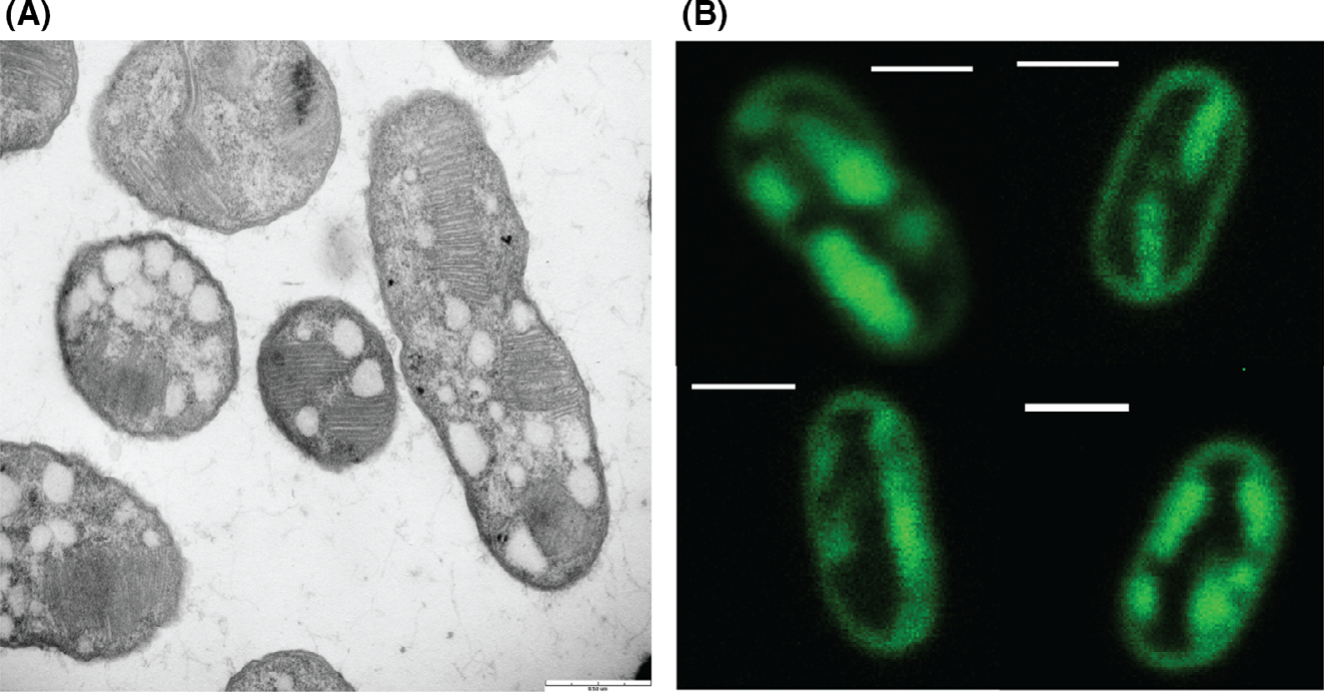
Electron micrograph (A) and fluorescence confocal images (B) of the type I methanotroph *M. alcaliphilum* comb. nov. 20Z. Membrane markers were the combination of osmium tetroxide plus uranyl acetate and FM 1‐43 respectively. ICMs appear as internal striations in the TEM image and present as fluorescent regions in the confocal images. The white circles in the TEM image are glycogen granules. Scale bar = 1 μm.

A method was developed for staining and imaging ICMs using the type I methanotrophic strain *M. alcaliphilum* comb. nov. 20Z as an initial model organism. The ultimate goal was to create a technique robust enough to be applicable to all methanotroph types. As a type I methanotroph, *M. alcaliphilum* comb. nov. 20Z produces its ICMs in stacked disc formations along the sides of the cell, which can be clearly seen via TEM (Fig. 1A). To visualize these structures via fluorescence, we first used the lipophilic styryl dyes FM 1‐43 (excitation maximum 479 nm/emission maximum 598 nm) and FM 4‐64 (ex 558/em 734). These dyes were chosen due to their high membrane specificity and quantum yield, as well as their low toxicity. They had also previously been used to image the membrane of *Escherichia coli* (Fishov and Woldringh, 1999) and sporulation in *Bacillus subtilis* (Pogliano *et al*., 1999; Sharp and Pogliano, 1999). Confocal microscopy imaging showed that the dyes labelled fluorescent regions within the cell (Fig. 1B), which were taken to be ICM structures stained by the lipophilic dye. Due to the limited resolution of the fluorescence microscope, it was not possible to distinguish individual membrane discs in the ICMs as seen in TEM images. Instead, the membrane stacks appear as solid fluorescent regions. This technique was applied to other type I methanotrophs with similar results. *Methyloprofundus sedimenti* and *Methylomonas methanica* S1 showed the same internal fluorescent regions (Fig. S1) as the fluorescent images of *M. alcaliphilum* comb. nov. 20Z. The cytoplasmic membrane is visibly labelled with the fluorescent probe and provides an outline of the image slice. Often, the signal intensity of the cytoplasmic membrane was less than that of the labelled ICMs, although we suspect that was due to the tightly packed ICMs having more membrane in that area and therefore more fluorescence label.

### Quantifying copper dependence of ICM coverage

**Figure 2:**
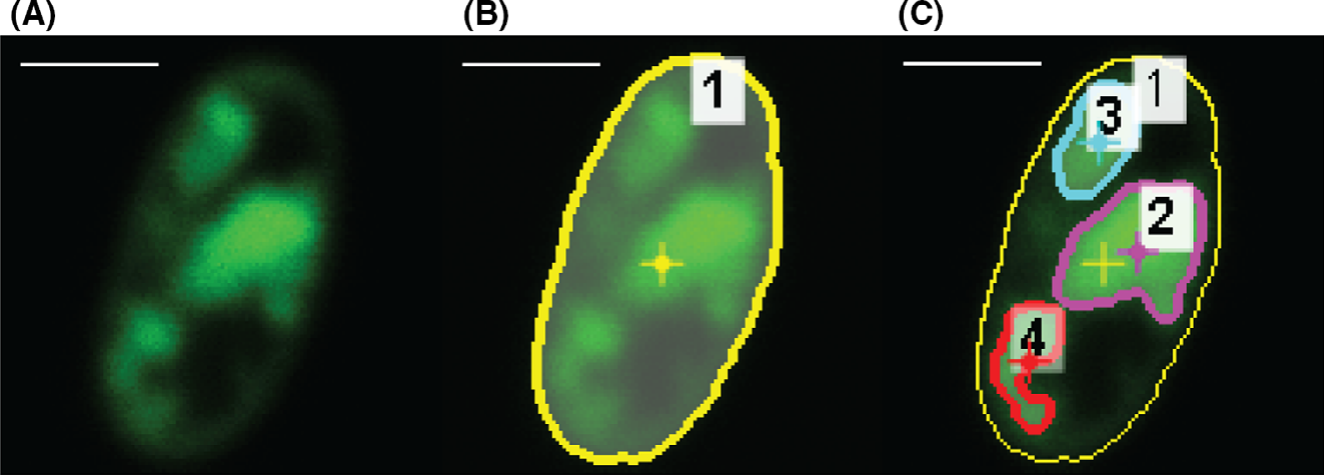
Sequence of events for ICM coverage analysis from fluorescence images. (A) Fluorescence image of an individual cell was isolated. (B) A region of interest (ROI) was created based on the periphery of the cell, providing an area encompassing the entire cell (C) ROIs corresponding to each fluorescent ICM subregion were determined from intensity data above a threshold within the whole cell ROI. The per cent of cellular area which is covered by ICMs was then calculated using areas of the ICM ROIs divided by the total cell area. Scale bar = 1 μm.

The extent of ICM formation on a cell by cell basis was quantified in confocal images by determining the per cent area occupied by the fluorescent regions of each cell (Fig. 2). Confocal images were taken of the rod‐shaped bacteria lying flat on the slide, so that the entire length was in the *x*–*y* plane. Images were taken with pinhole settings such that the sections of the cell envelope on the top and bottom of the cell along the *z*‐axis cell fell outside of the focal plane. This ensured that primarily the cytoplasmic area was imaged in the selected image slice and that fluorescent signal would not come from the membrane surrounding the cytoplasm. Assuming the compression of each membrane stack is similar, this measurement can be used as a proxy for the amount of ICMs and therefore lipid content, within the cell. Because these data were collected on a single‐cell basis, the resulting per cent of coverage can be analysed as a distribution of cells from the same sample to examine heterogeneity or as an aggregation of data.

**Figure 3:**
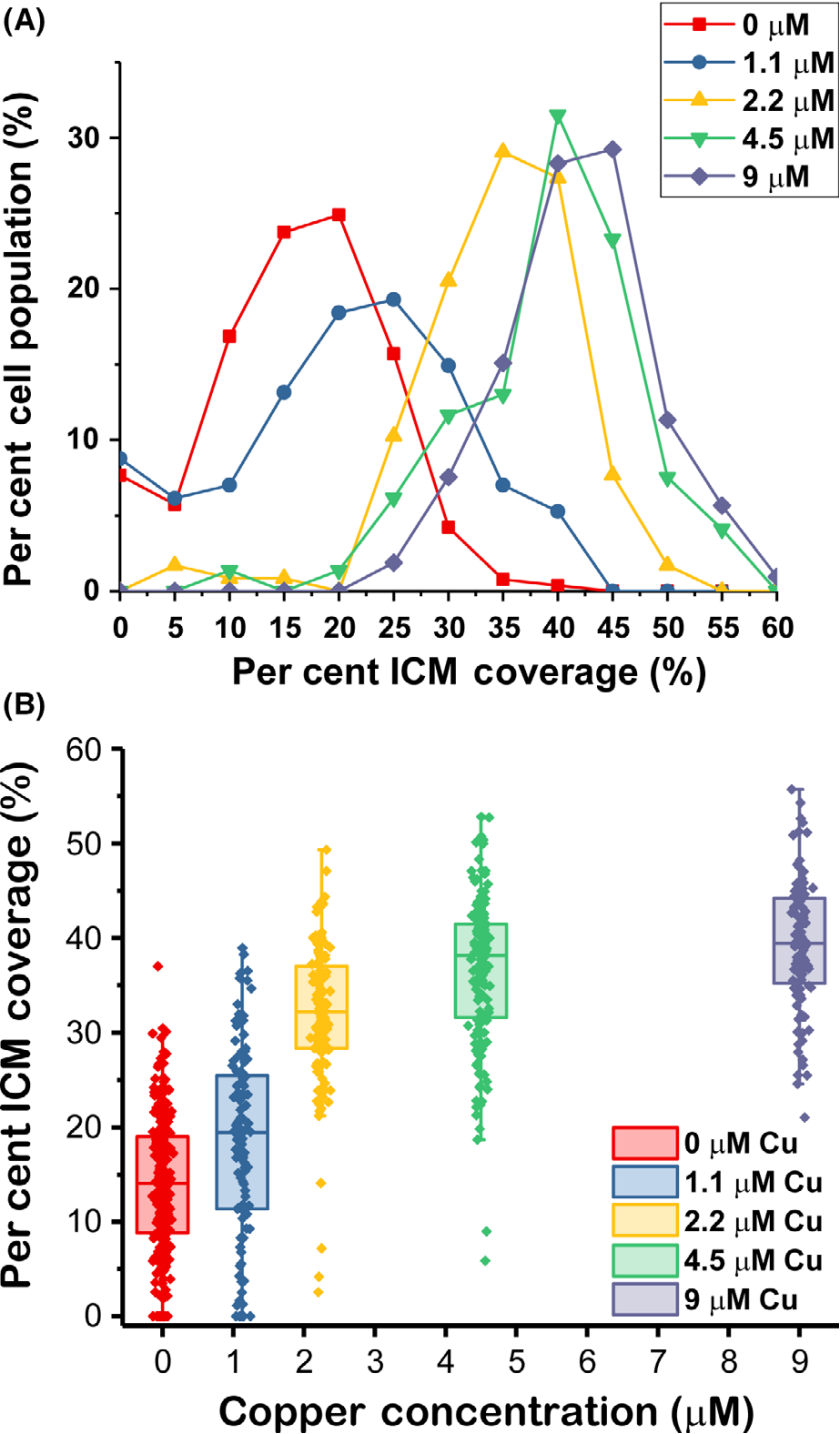
Change in per cent ICM coverage in response to copper availability. Data come from *M. alcaliphilum* comb. nov. 20Z cultures grown in the presence of varying amounts of copper, with methanol (0.2% V/V) as the carbon source. More than 100 cells were imaged for each condition. ICM coverage increased with copper availability, indicating a clear dosewise response up until the 4.5 μM copper condition. (A) Individual cells sorted into histograms in terms of per cent of total population imaged. (B) Individual cell measurements represented by dots represent while the box gives the range from the 25th to 75th percentile. The bar represents the median measurement, and the whiskers extend to 1.5 times the interquartile range.

The dependence of ICM formation on copper availability has been shown previously in many methanotrophs (Collins *et al*., 1991; Brantner *et al*., 1997). In general, available copper promotes ICM formation and organization, while copper limitation likewise reduces ICM presence. Many methanotroph strains lose ICMs entirely in the absence of copper, leading this phenomenon to be referred to commonly and the “copper switch” (Semrau *et al*., 2013). To assess the validity and adaptability of the labelling of ICMs with FM dyes, ICM coverage with respect to copper concentration in the growth media was tracked in *M. alcaliphilum* comb. nov. 20Z (Fig. 3). Copper concentrations from 0 to 9 μM were analysed with more than 100 cells under each growth condition. To maintain a consistent carbon source across all conditions, all cells were grown in methanol (0.2% V/V)‐containing media. There was no significant difference (*P*‐value = 0.919 for a two‐tailed *t*‐test) in per cent ICM coverage between *M. alcaliphilum* comb. nov. 20Z cells grown with methanol or methane as the carbon source (Fig. S2) with 4.5 μM copper.

As expected, the ICM coverage increased as the copper concentration was raised. Even in copper‐free media, a small amount of ICM formation still persists, perhaps either due to trace amounts of copper carried over from previous cultures or other physiological reasons. ICM formation in *M. alcaliphilum* comb. nov. 20Z, as determined by the average ICM coverage, increased until it plateaued at a copper concentration of 4.5 μM (Fig. 3). ICM formation reached a maximum capacity at this point, even though the cells can withstand larger copper concentrations without an inhibition of growth. Cells grown with 9 μM copper showed minimal differences in the distribution of ICM coverage from that of the 4.5 μM cultures. The cells are not completely filled with ICMs at this point, although space may be needed for other components such as the nucleoid.

These results match the trends commonly seen in membranes at varying copper levels of other methanotrophs and are consistent with our own total lipid analysis of *M. alcaliphilum* comb. nov. 20Z cells grown with varying copper concentrations. The per cent total lipid was measured as described in Experimental Procedures and determined to be 5.6 ± 1.1% for cells grown with 0.0 μM copper and methanol as the carbon source (not possible to grow *M. alcaliphilum* comb. nov. 20Z on methane in copper‐free media as they do not express soluble methane monooxygenase). Like our fluorescence measurements, the amount of lipid increased with increasing copper concentrations in the media to the 4.5 μM copper concentration at which point it plateaued. This occurs if methanol or methane was used as the carbon source. Per cent total lipid for 2.2, 4.5 and 9.0 μM copper were 15.3 ± 3.6%, 19.2 ± 2.3% and 19.7 ± 2.3%, respectively, for methanol‐grown cells and 13.8 ± 5.2%, 20.3 ± 4.2% and 19.4 ± 4.2%, respectively, for methane‐grown cells. These numbers differ from the per cent coverage fluorescence measurements since they are based on biomass and lipid weights instead of pixel area above a threshold, although the trend is consistent and they show there is no difference between using methanol and methane as the carbon source. The closest comparison to this type of coverage analysis was done in *Methylomicrobium album* BG8, although in that case cells were classified into groups based on the extent of ICM development at varying copper concentrations (Brantner *et al*., 1997). The researchers found that most cells grown with < 0.74 μM copper had poorly developed ICMs or no ICMs, at 1 μM copper, 60% of cells had more structured ICMs, and above 4 μM copper, 90% of cells either had more structured ICMs or were packed with ICMs. We believe these results are consistent with the trends we found. Also, based on our TEM images and fluorescence images of *M. alcaliphilum* comb. nov. 20Z from the same sample, the areas covered by ICMs in the both TEM and confocal images were determined to be the same (Fig. S3). We found this to be true for both cells grown in 0.0 and 4.5 μM copper.

### Live imaging of fluorescently labelled ICMs in type I methanotrophs

**Figure 4:**
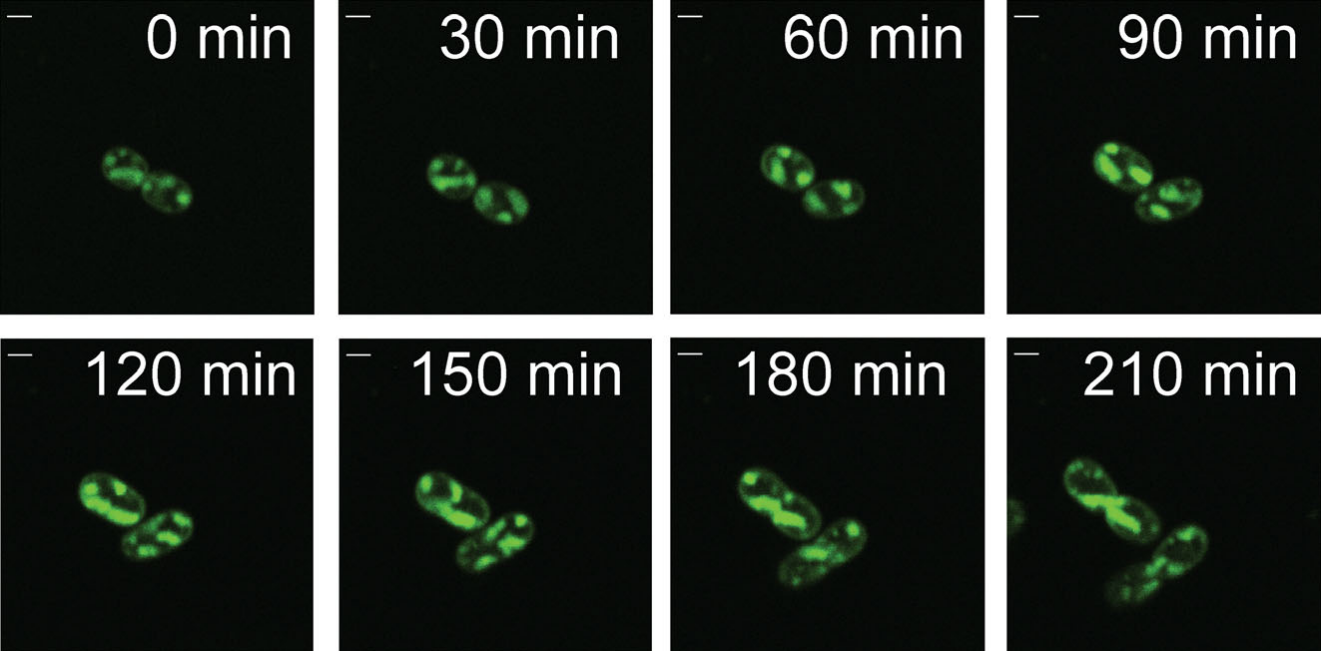
Time‐lapse fluorescence images at 30‐min intervals of *M. alcaliphilum* comb. nov. 20Z stained with Mitotracker Green FM and grown at 30°C with methanol (0.2% V/V) as the carbon source and 4.5 μM copper in the media. Cells elongated and divided as normal, with changes detected in ICM position and coverage during growth. Scale bar = 1 μm.

In addition to collecting static images at a single time point to monitor ICM coverage differences between separate cultures, fluorescently labelling ICMs allow for the tracking of ICM changes in a single cell over long periods of time. Unfortunately, although the FM 1‐43 dye itself is not cytotoxic, long laser exposure times with this dye caused cell death, presumably due to local heating, reactive oxygen species generation, membrane fluidity changes or a combination of these factors. Mitotracker Green FM (MTG) is a mitochondria‐specific dye that has been shown to stain lipids in Gram‐negative bacteria (Schneider *et al*., 2007). Although it did not provide as good a signal‐to‐noise ratio as the FM dyes, it successfully labelled ICM in *M. alcaliphilum* comb. nov. 20Z. ICM shape and coverage were tracked at 30‐min intervals for cells grown at 30°C in a culture dish with a poly‐l‐lysine‐coated coverslip as the base (Fig. 4) and methanol (0.2% V/V)‐containing media with 4.5 μM copper. The per cent ICM coverage in the cells tracked over time varied from 20% to 55%, as found in the bulk growth cultures. Doubling times of cells monitored over time on the microscope were consistent with those from cell culture under the same media and temperature conditions.

### Examining possible ICM connectivity to cytoplasmic membrane in type I methanotrophs

It has been previously suggested that the ICMs of type I methanotrophs exist as invaginations of the cytoplasmic membrane (Whittenbury *et al*., 1970; De Boer and Hazeu, 1972; Brantner *et al*., 2000, 2002). Results we have seen through our fluorescent images seem to be consistent with this possibility. The FM dyes are membrane impermeable, meaning that it is not possible for them to cross‐over to label membrane components completely isolated in the cytoplasm. They traditionally were used to track vesicle formation in neurons (Stevens and Williams, 2000), although they had also been used in bacteria to look at sporulation in *Bacillus subtilis* (Pogliano *et al*., 1999; Sharp and Pogliano, 1999) and the connectivity of cytoplasmic and thylakoid membranes (Schneider *et al*., 2007). The ability of the FM dyes to successfully label the ICMs of *M. alcaliphilum* comb. nov. 20Z and other type I methanotrophs would suggest that the ICMs are connected to the cytoplasmic membranes, are invaginations of that membrane or that there may be some cycling from the cytoplasmic membrane to ICMs.

We attempted to validate the ICM connectivity to the cytoplasmic membrane with fluorescence recovery after photobleaching (FRAP). ICM fluorescence in FM 1‐43‐stained *M. alcaliphilum* comb. nov. 20Z was photobleached, and fluorescence recovery was monitored for 30 s. The only way recovery of ICM fluorescence could occur if the ICMs were directly attached to the cytoplasmic membrane, allowing FM 1‐43 to diffuse through this membrane to the ICM. Cells were resuspended in media free of FM 1‐43 dye after initial labelling to ensure that recovery was from the cytoplasmic membrane and not free dye in the media. Fluorescence recovery of bleached ICM was seen, taking 6 s on average to reach a maximum within the 30‐s window of observation (Fig. S4). The timescale suggests that the recovery is more likely due to connectivity than cycling of membrane.

**Figure 5:**
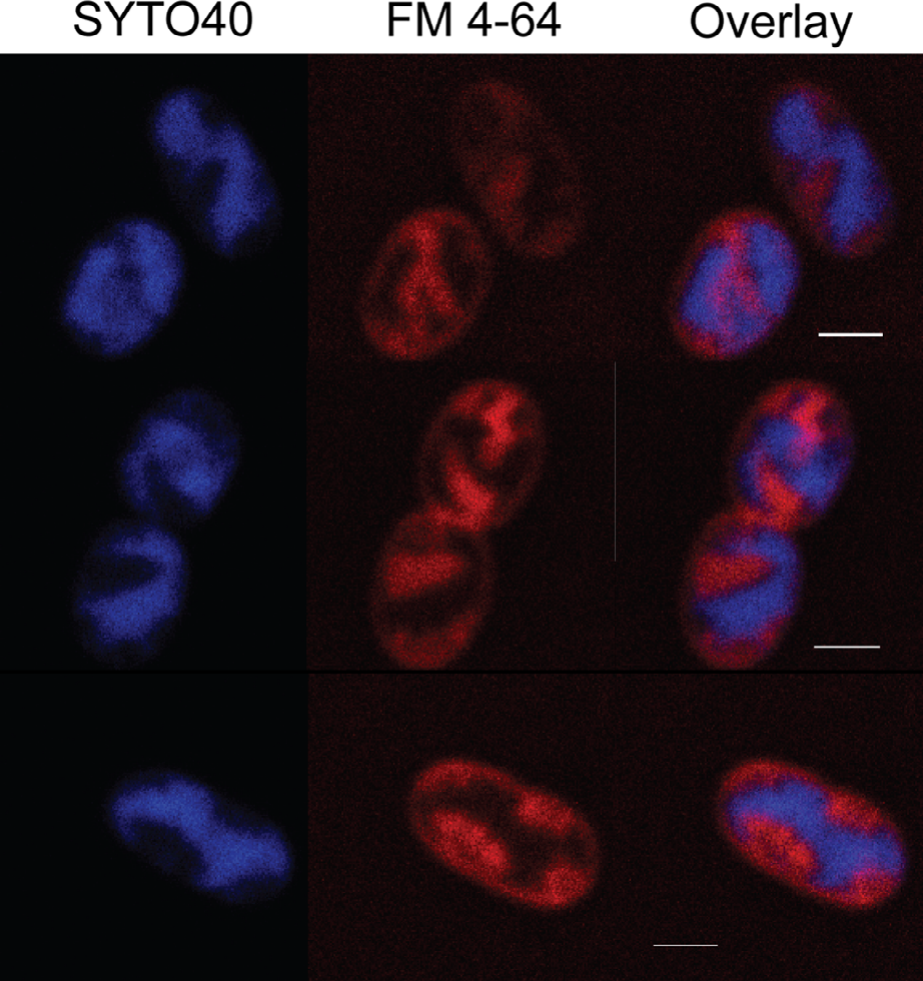
Fluorescence Images of *M. alcaliphilum* comb. nov. 20Z costained with FM 4‐64 (red) and SYTO40 (blue). Membrane fluorescence signals have strong anticorrelation with cytoplasmic signals. Scale bar = 1 μm.

**Figure 6:**
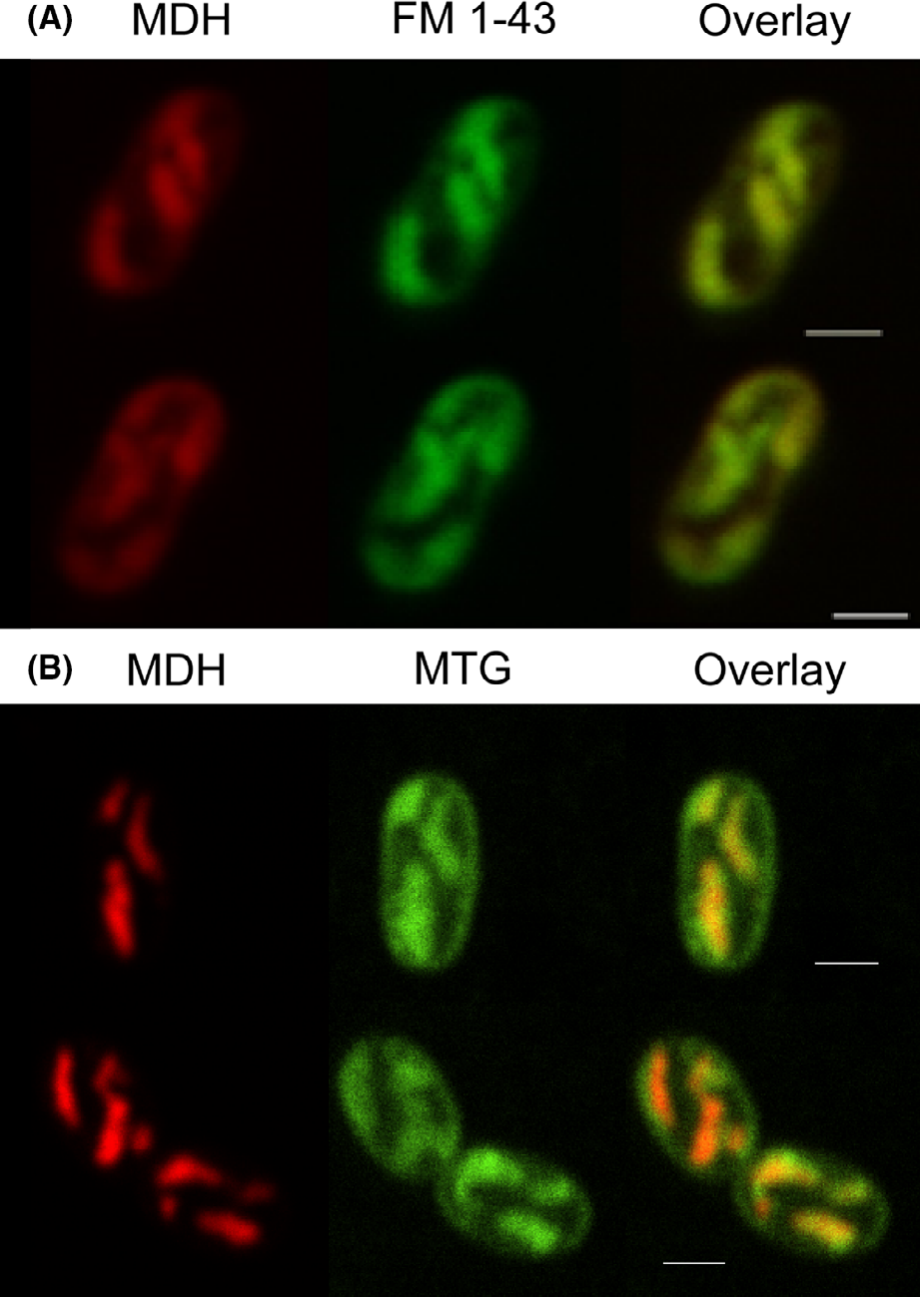
*M. alcaliphilum* comb. nov. 20Z expressing mCherry labelled methanol dehydrogenase (MDH, red, left) and stained (green, centre) with either (A) FM 1‐43 or (B) MitoTracker Green (MTG). An overlay of the two channels is shown to the right. The MxaF‐mCherry fluorescence appears colocalized with ICMs, potentially due to ICMs being invaginations of the cytoplasmic membrane. Scale bars represent 1 μm.

Costaining of *M. alcaliphilum* comb. nov. 20Z cells with FM 4‐64 and the nucleic acid binding SYTO40 as a cytoplasmic marker was also performed. ICM and cytoplasmic signals were seen to be anticorrelated (Fig. 5). Because no cytoplasmic signal is seen within the ICM regions, it indicates that the probe is being excluded from the lumen of the ICM, although this does not necessarily exclude the ICMs being isolated membrane stacks in the cytoplasm. Therefore, fluorescent fusion proteins between mCherry and the periplasmic enzyme methanol dehydrogenase (MDH) were created to look at the localization in the cell relative to ICMs. Both MxaF‐mCherry and MxaI‐mCherry were made, although the cells expressing the MxaF‐mCherry variant had better signal to noise. Upon expression in *M. alcaliphilum* comb. nov. 20Z, mCherry signal can be seen to colocalize with MTG signal corresponding to ICMs (Fig. 6). This is consistent with past immunolabelling experiments showing MDH localized with ICMs in TEM images (Brantner *et al*., 2002). The localization of the periplasmic MDH to the ICM would be consistent with ICMs being invaginations of the cytoplasmic membranes or require that a periplasmic‐like environment be maintained in the lumen of vesicular ICMs in the cytoplasm. At a minimum, it confirms the labelling of ICMs with the fluorescent probes.

## Discussion

The development of this fluorescence‐based method for labelling and identifying ICMs is a valuable tool for studying ICMs. We have shown that it is possible to follow the copper dependence of ICM formation and couple these measurements with other labels. While the fluorescence images are not as detailed as TEM or cryo‐EM images of the membrane structures, the same per cent ICM coverage can be calculated from them as is determined by TEM images. There are other advantages to this approach as well. The first is that it may be accessible to more researchers due to the associated costs of the EM methods. Using a fluorescence microscope could be a more cost‐effective method as they are less expensive and available on many campuses in core facilities. This could be particularly useful for rapidly assessing different strains to determine the extent of ICM formation, particularly when creating mutants. Also, the use of fluorescent labels can be used to follow ICM changes in the same individual cell over time for hours. This can be coupled with fluorescently labelled proteins of interest or other single‐cell approaches, such as tracking changes in an individual cell as the copper concentration changes.

We found that FM dyes stain the inner membrane and ICMs of type I methanotrophs completely and were the best overall probe if not tracking a cell over time. This differs from some other studies of Gram‐negative bacteria where the FM dye only stains the outer membranes and does not label the inner membrane (Pilizota and Shaevitz, 2012; Zupan *et al*., 2013). The colocalization of the fluorescently tagged MDH with FM 1‐43 in *M. alcaliphilum* comb. nov. 20Z is only possible if the dye does reach the inner membrane. In addition, FM 1‐43 results match MTG labelling and the TEM per cent ICM coverage results. It is possible that peptidoglycan structural differences could account for the differences in labelling (Rogers and Wright, 1987).

In type I methanotrophs specifically, the incubation time used is important to ensure proper staining of the cells. When *M. alcaliphilum* comb. nov. 20Z, *Methyloprofundus sedimenti* and *Methylomonas methanica* S1 were stained with FM dyes for less than 30 min, ICMs either did not appear or characteristically had poor signal‐to‐noise, making quantification impossible (Fig. S5). This differs greatly from the manufacturer's recommendations, which state that cells should be stained with a lower dye concentration than what we used and viewed immediately after staining for best results. This recommendation can be attributed to the fact that FM dyes traditionally were used to track vesicle formation in neurons (Stevens and Williams, 2000). Staining longer than 1 hour showed no significant increase in the quality of images. The extended staining time may be due to the time required for the dye to reach the inner membrane, equilibrate throughout the highly compressed, invaginated ICM stacks in type I methanotrophs or for the cycling of labelled cytoplasmic membrane to isolated, internalized ICMs because of the impermeability of the FM dyes. The membrane permeable dye MTG stains ICMs in a fraction of the time, although we found it typically had a lower signal‐to‐noise ratio, so it would only be preferred when live cell tracking was necessary.

From a throughput perspective, an intensity‐based method for determining ICM content would be preferable over the area‐based method presented here since it would enable large numbers of cells to be analysed by flow cytometry. Caution should be used though as intensity‐based measurements were found to be somewhat unreliable. Some cell with greater ICM coverage showed less overall fluorescence intensity than cells with less ICM area. Cell‐to‐cell variability in dye uptake, compressibility of the ICM stacks or self‐quenching of the membrane probe could possibly explain the variability.

We also found that copper concentration was the determining factor in the extent of ICM formation. Using methane or methanol as a carbon source did not affect the amount of ICMs, as determined by total lipid analysis and fluorescence imaging. This may seem counterintuitive since copper is important for the active site of pMMO, and if the enzyme is being bypassed by directly growing on methanol, why make such extensive membrane structures? Considering the native environment where methane may not continuously be available to cell, it could be that it is advantageous to maintain membrane structures if copper is available for pMMO, particularly if ICMs help concentration methane in the cell (Lawton and Rosenzweig, 2016). It certainly warrants further study.

While the described fluorescence approaches can provide some insights as to structural details about ICMs, they cannot provide the visualization of the membrane structures like the EM techniques. To completely address the connectivity question of ICMs to the cytoplasmic membrane, it is likely that cryo‐EM or other fast‐sample preparations for TEM will need to be applied, as has been done in some cases (Kip *et al*., 2011; van Teeseling *et al*., 2014; Tavormina *et al*., 2017). We see the marriage of fluorescence microscopy and EM techniques (Biel *et al*., 2003; Giepmans, 2008; Boedeker *et al*., 2017) forwarding our understanding of the structure/function relationship of ICMs, much as has been done with cristae and mitochondria (Cogliati *et al*., 2016). In that way, the fluorescence approaches described here provide a valuable tool in this area of research.

## Experimental procedures

### Bacterial strains and growth conditions

Type I methanotroph strains used in this study were as follows: *Methylomicrobium alcaliphilum* comb. nov. 20Z, *Methyloprofundus sedimenti* and *Methylomonas methanica* S1. These strains were grown in 25 ml cultures within 250 ml gas tight serum bottles, using nitrate mineral salts medium (Whittenbury *et al*., 1970) modified to contain only 0.2 g MgSO_4_×7H_2_O and with a salinity (NaCl) of 7.5% (*M. alcaliphilum* comb. nov. 20Z), 20% (*M. sedimenti*) or 0% (*M. methanica* S1). All methanotrophic strains were grown at 30°C with shaking (200 r.p.m.) except for *M. sedimenti* which was grown at room temperature (25°C) without shaking. Carbon sources for the methanotrophs were either methane (1:2.25 methane:air headspace ratio) or methanol (0.2% V/V). *E. coli* S17‐1 was grown in LB (Luria‐Bertani) Broth, Miller (BD Biosciences, Sparks, MD, USA) at 37°C with shaking (200 r.p.m.). For copper‐limited experiments, cells were grown in 5 ml cultures in disposable plastic culture tubes to prevent copper carry‐over from glassware. Media for copper‐limited studies was prepared and stored in glassware that had been rinsed at least three times with 3 M nitric acid and rinsed with ethanol before final rinses with ultrapure H_2_O.

### Methanol dehydrogenase fluorescent fusion protein

The creation of a c‐terminal fluorescent fusion protein for MDH was done using pCM66 plasmid (Marx and Lidstrom, 2001) as a starting point and inserting the genes to generate the fusion protein. Plasmids for a fusion protein for both the large subunit (*mxaF*, NCBI Reference Sequence: WP_014149867.1) and small subunit (*mxaI*, NCBI Reference Sequence: WP_014149864.1) of *M. alcaliphilum* comb. nov. 20Z MDH were created (pKW1: MxaF‐mCherry and pKW2: MxaI‐mCherry). Inserts included genes encoding the promoter region for *mxaF* (500 bp region upstream of *mxaF*), the respective subunit and mCherry (Shaner *et al*., 2004) as a fluorescent reporter. Plasmid assembly was done with the NEBuilder HiFi DNA Assembly Cloning Kit (New England Biolabs, Ipswich, MA, USA), with PCR primers being generated according to the NEBuilder assembly web tool, with slight modifications to increase PCR yield. PCR was performed to generate linearized inserts with matching overhangs to promote complementarity. Upon completion of PCR, the NEBuilder assembly solution was used to assemble the plasmids based on end complementarity.

Transformation was done via conjugation with a donor strain, *E. coli* S17‐1. Chemically competent *E. coli* S17‐1 cells were made by treatment with rubidium chloride as commonly described elsewhere (Green and Rogers, 2013). These competent cells were then transformed with the plasmid of interest via heat shock (Hanahan *et al*., 1991) to create the donor strain. This strain was then grown on standard LB plates for later use. At the same time, the acceptor strain (*Methylomicrobium alcaliphilum* comb. nov. 20Z) was grown on plates with modified NMS media according to their standard growth conditions. Biomass from both the donor and acceptor strains were then streaked together on mating plates with modified NMS media supplemented with a sufficient amount of LB (100 ml l^−1^) to satisfy *E. coli*. Mating plates were incubated with methane at 30°C for between 24 h and 48 h. Aggregate biomass was then spread onto modified NMS plates containing selection antibiotic (kanamycin, 50 μg ml^−1^) and no LB. These plates were again incubated with methane. Single colonies were selected within 3 weeks and cultured as normal in the presence of antibiotic.

### Sample preparation

Cells for imaging were grown between 0.2 and 0.8 OD_600_. Cells grown in differing copper conditions were subcultured a minimum of three times at the appropriate copper concentration before imaging. For static fluorescence imaging of membranes, cell was stained with either FM 1‐43 (ThermoFisher/Invitrogen, Waltham, MA, USA, green, excitation: 488 nm) or FM 4‐64 (red, excitation: 561 nm), to a final concentration of 5 μg ml^−1^ in growth media. For long‐term live imaging on the microscope, Mitotracker Green FM (ThermoFisher/Invitrogen, Waltham, MA, USA, green, excitation: 488 nm) at a final concentration of 1 μM in the media was used. A final concentration of 1 μM SYTO40 (ThermoFisher/Invitrogen, Waltham, MA, USA, blue, excitation: 405 nm) was used for nucleic acid staining of cells. All cells were stained at their growth temperature for the following times: 1 h for the FM dyes, 30 min for MTG and 15 min for SYTO40. Costaining with FM 4‐64 and SYTO40 was carried out by adding each dye sequentially and allowing it to incubate for the full duration before the next dye was added.

For TEM imaging, cells were first fixed with freshly made 1% glutaraldehyde for 1 h at room temperature (25°C). Cells were then stained with 1% osmium tetroxide and incubated at 4°C overnight. The following day cells were stained with 2% uranyl acetate at 25°C for 1 h, harvested by pelleting in agarose and dehydrated overnight by acetone addition. Finally, samples were infiltrated and embedded with Poly/Bed 812 plastic before thin slices were cut from the block.

### Imaging

Fluorescence images were taken on a Nikon A1 confocal microscope with the Elements software package using a 100× Plan Apo λ (NA 1.45) oil objective. Excitation of fluorescence probes was made with solid‐state lasers and their emission isolated by bandpass filters as follows; SYTO40: 405 nm excitation, 425–475 nm filter, MTG: 488 nm excitation, 500–550 nm filter, FM 1–43: 488 nm excitation, 521.5–554.5 nm filter, FM 4–64: 488 nm or 561 nm excitation and 575–625 nm filter. Short‐term imaging was done on glass slides coated with 0.01% poly‐l‐lysine (Sigma‐Aldrich, St. Louis, MO, USA). Imaging over time was performed with an Attofluor cell chamber (ThermoFisher/Invitrogen, Waltham, MA, USA) using a standard poly‐l‐lysine‐coated coverslip as a base. For long‐term experiments, the sample was kept at a constant growth temperature of 30°C by an Okolab microscope cage incubator. TEM imaging was performed by the Ohio State University Ohio Agricultural Research and Development Center's Molecular and Cellular Imaging Center in Wooster, Ohio.

### FRAP measurements

All FRAP measurements were performed on a Nikon A1 confocal laser scanning microscope. Individual cells were chosen, and a portion of their ICMs was selected for photobleaching using the region of interest (ROI) tool on the Nikon Element software package. After initially imaging the entire cell, an intense scan was focused within the ROI for 1 s to photobleach the fluorescent label in this region. Then, subsequent images were taken of the entire cell at 1‐s intervals.

### Data analysis

Region of Interest (ROI) generation was performed using the Nikon Elements software package. For each cell, a region of interest surrounding the whole cell was created from the cell outline (Fig. 2B). Subsection ROIs were then determined for each individual instance on internal fluorescence within the same cell from intensity data above a threshold (Fig. 2C). An area measurement for each region was calculated based on the pixel count for each ROI. From these data, a measurement of ICM area coverage was evaluated using the formula:

$$\%\;\text{ICM}=\frac{\sum_{i=1}^{n}a_{i}}{a_{t}}\times 100\%$$

where *a*_*i*_ is the area of a subsection ROI for an ICM and *a*_*t*_ is the total cell area.

### Per cent total lipid analysis

Procedure based on method by Bligh and Dyer (Bligh and Dyer, 1959; Breil *et al*., 2017). Cells were grown until they reach exponential phase OD_600_ = 0.3–0.8. A total of 50 ml of each culture was harvested by centrifugation at 10 000–20 000 × *g* at 4°C for 15 min. Pellets were washed with 70% ethanol, and the cell pellets were stored at −20°C, with 0.5–1.0 ml of nanopure water. After solidification, cell pellets were lyophilized under vacuum, to get the dried biomass and weighed. After drying in a glass tube, the biomass was resuspended in 2 ml of deionized water and 2.5 ml of methanol and 1.25 ml of dichloromethane were further added. The solutions were mixed and sonicated for 1 h. Then, 5 ml of deionized water and 5 ml dichloromethane were added with the tube, which was vortexed to thoroughly mix the solution and then incubated at −20°C overnight. Samples were centrifuged for 10 min at 3000 × *g*, and the organic layer was transferred to a 15‐ml baked glass vial using Pasteur pipette. The total lipid extract was evaporated under N_2_ and dried before analysis. The per cent total lipid was equal determined by the following formula: % total lipid = (lipid extract weight/total biomass weight)*(volume of organic layer/volume of organic layer evaporate).

## Conflict of interest

None declared.

## Supporting information


**Fig. S1.** Fluorescence images of the type I methanotrophs *M. sedimenti* and *M. methanica* S1 stained with FM 1‐43.
**Fig. S2.** *M. alcaliphilum* 20Z grown in modified nitrate mineral salts medium with methane (1:2.25 methane:air headspace ratio) or methanol (0.2% V/V) showed no significant difference in the distribution of the ICM coverage based on the carbon source for cells grown with 4.5 μM copper.
**Fig. S3.** Comparison of percent ICM coverage measured in *M. alcaliphilum* 20Z using TEM images and confocal image with FM 1‐43 as the membrane stain for both the 0 μM and 4.5 μM copper growth conditions.
**Fig. S4.** FRAP experiment performed on *M. alcaliphilum* 20Z supporting ICM cytoplasmic membrane attachment.
**Fig. S5.** Poor FM 1‐43 staining due to short incubation with dye. *M. alcaliphilum* 20Z were stained with FM 1‐43 and incubated for 15 min.
