# Supplementary material for: Fluorescence‐based analysis of the intracytoplasmic membranes of type I methanotrophs
Source: Microb Biotechnol. 2019 Jul 1;12(5):1024–33. doi: 10.1111/1751-7915.13458 (PMC6680624; doi:10.1111/1751-7915.13458)
Supplement: Supplementary file 1 — Fig. S1. Fluorescence images of the type I methanotrophs M. sedimenti and M. methanica S1 stained with FM 1‐43. Fig. S2. M. alcaliphilum 20Z grown in modified nitrate mineral salts medium with methane (1:2.25 methane:air headspace ratio) or methanol (0.2% V/V) showed no significant difference in the distribution of the ICM coverage based on the carbon source for cells grown with 4.5 μM copper. Fig. S3. Comparison of percent ICM coverage measured in M. alcaliphilum 20Z using TEM images and confocal image with FM 1‐43 as the membrane stain for both the 0 μM and 4.5 μM copper growth conditions. Fig. S4. FRAP experiment performed on M. alcaliphilum 20Z supporting ICM cytoplasmic membrane attachment. Fig. S5. Poor FM 1‐43 staining due to short incubation with dye. M. alcaliphilum 20Z were stained with FM 1‐43 and incubated for 15 min. [file MBT2-12-1024-s001.pdf]

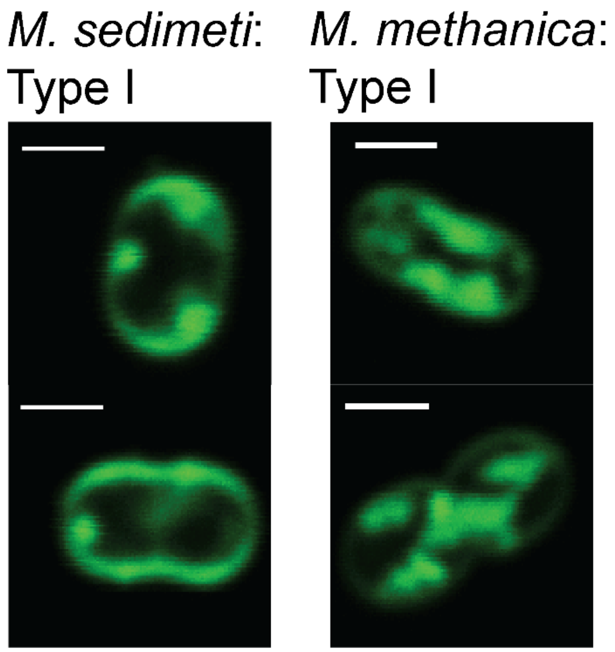

Fig. S1. Fluorescence images of the type I methanotrophs *M. sedimenti* and *M. methanica* S1 stained with FM 1-43. Both strains are consistent with characteristics of the previously discussed type I strain, *M. alcaliphilum* 20Z. Scale bar = 1 µm.

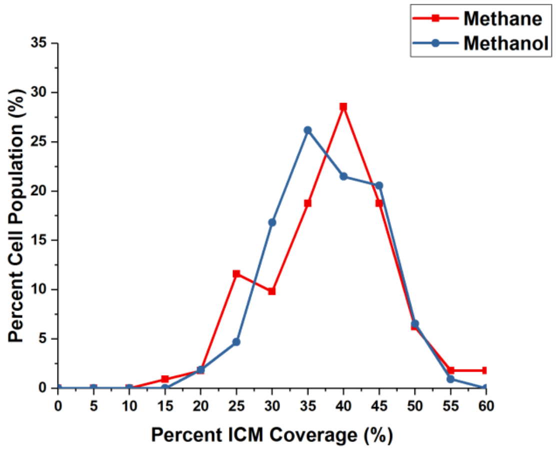

Fig. S2. *M. alcaliphilum* 20Z grown in modified nitrate mineral salts medium with methane (1:2.25 methane:air headspace ratio) or methanol (0.2% V/V) showed no significant difference in the distribution of the ICM coverage based on the carbon source for cells grown with 4.5 μM copper. For a two-tailed student-t test, the p-value equals 0.919, indicating the null hypothesis holds true. Scale bar = 1 μm.

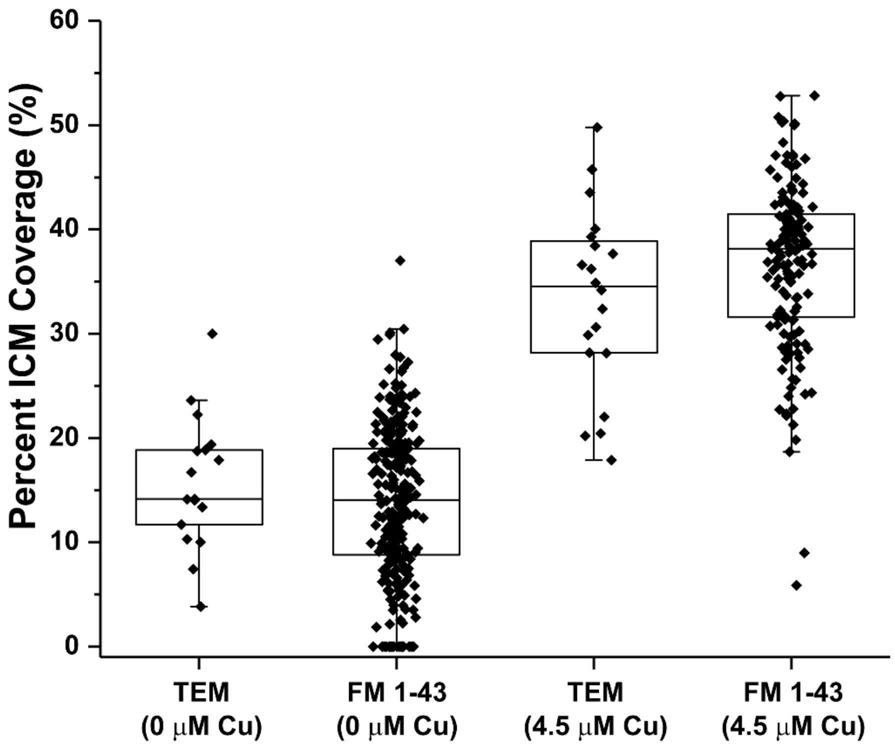

Fig. S3. Comparison of percent ICM coverage measured in *M. alcaliphilum* 20Z using TEM images and confocal image with FM 1-43 as the membrane stain for both the 0 μM and 4.5 μM copper growth conditions. No significant difference found between the two measurement approaches. Dots represent individual cell measurements while the box gives the range from the 25th to 75th percentile. The bar represents the median measurement and the whiskers extend to 1.5 times the interquartile range.

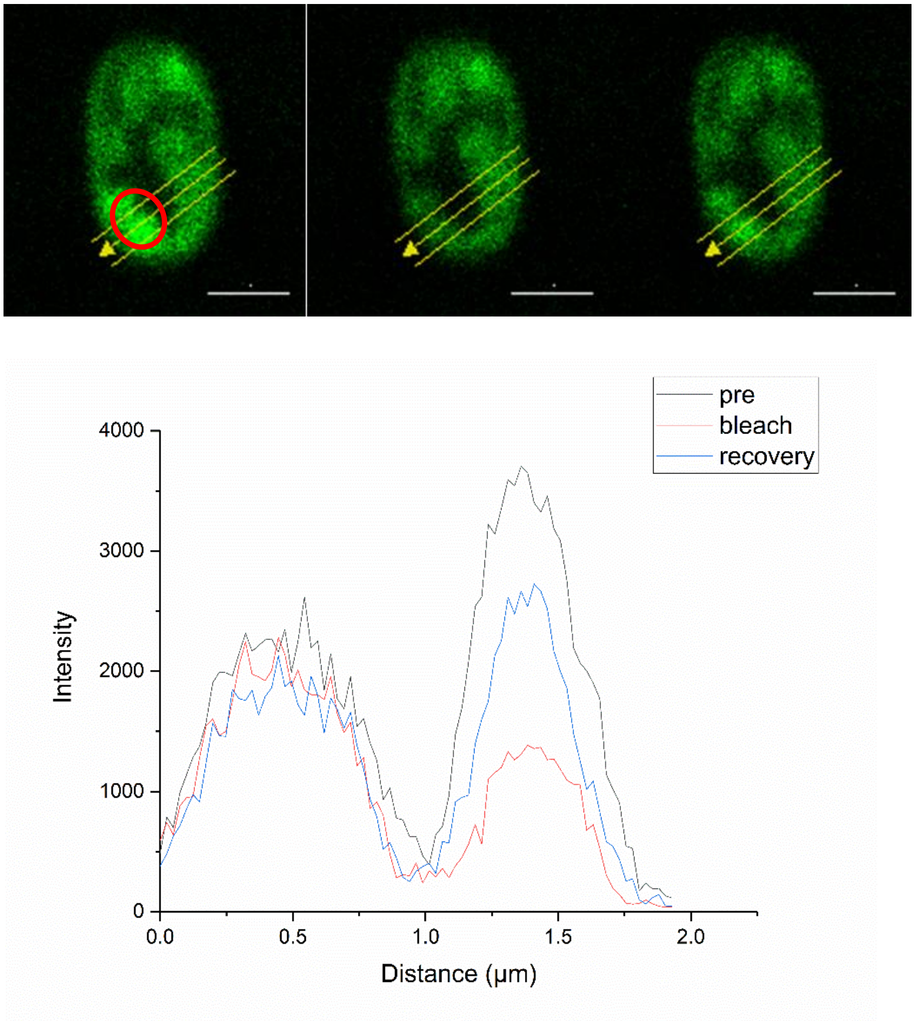

Fig. S4. FRAP experiment performed on *M. alcaliphilum* 20Z supporting ICM cytoplasmic membrane attachment. The above fluorescence images show a cell before photobleaching (pre, left), immediately following a 1 s photobleaching event (bleach, center), and after maximum recovery had been reached after 6 s of imaging in 1 s intervals after the bleach (recovery, right). Bleach area is indicated by the red circle. Intensity data along the arrow is plotted below for each image. Overall intensity at recovery is lower than the prebleach condition because a portion of the dye has been photobleached. Scale bars represent 1 μm.

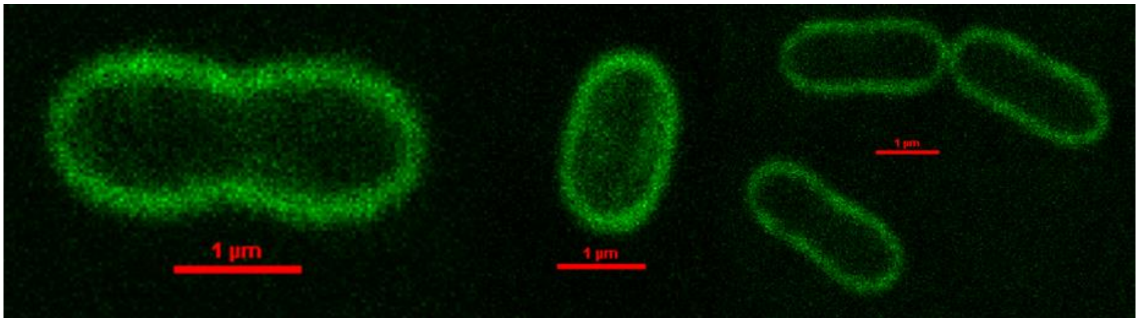

Fig. S5. Poor FM 1-43 staining due to short incubation with dye. *M. alcaliphilum* 20Z were stained with FM 1-43 and incubated for 15 min. ICMs are difficult to distinguish from background in contrast to cells that were incubated with stain for 1 hour. Scale bar represent 1 µm.
